# Human pluripotent stem cell engineering with CRISPR–Cas9 for Parkinson’s disease

**DOI:** 10.1038/s12276-026-01679-2

**Published:** 2026-04-10

**Authors:** Seung Bin Park, Ji-Soo Kim, Yuri Ha, Min Seong Kim, Tae Wan Kim

**Affiliations:** 1 Department of New Biology, Daegu Gyeongbuk Institute of Science and Technology, Daegu, Republic of Korea; 2 Department of Integrative Bioscience and Biotechnology, Institute of Bioscience, Sejong University, Seoul, Republic of Korea; 3 Department of Biomedical Science and Engineering, Daegu Gyeongbuk Institute of Science and Technology, Daegu, Republic of Korea; 4 New Biology Research Center, Daegu Gyeongbuk Institute of Science and Technology, Daegu, Republic of Korea

**Keywords:** Stem-cell differentiation, Parkinson's disease, Stem-cell therapies, Disease model

## Abstract

Parkinson’s disease (PD) entails loss of substantia nigra dopamine (DA) neurons and α-synuclein pathology. Currently, no effective disease-modifying therapies have been developed. Human pluripotent stem cells (hPS cells) can generate DA neurons on scale, enabling human genetic PD modeling of mitochondrial, lysosomal and synaptic connection failure that leads to DA neuron degeneration. Clustered regularly interspaced short palindromic repeats (CRISPR) extends this human model by providing causal, isogenic interrogation and transcriptional regulation of PD genes and reporter knock-ins that support purification and high-content screening. hPS cell-based DA cell grafts can restore motor function yet face >90% acute cell death and product heterogeneity in vivo post implantation. CRISPR enabled not only an in vivo cell survival screen to identify the cell death regulators but also a reporter-guided enrichment of DA neurons and chemogenetic control of grafted DA cell function in vivo. Here we summarize this progress and outline a practical road map to accelerate the development of precise human models and advanced hPS cell-based cell therapies for PD.

## Enhancing neuron survival in Parkinson’s disease models

Parkinson’s disease (PD) is a common brain disorder affecting millions worldwide, mainly due to the loss of dopamine-producing neurons. Researchers are exploring new ways to model and treat PD using human pluripotent stem cells (hPS cells) and CRISPR gene-editing technology. hPS cells can be turned into dopamine neurons, which are crucial for studying PD. CRISPR allows precise changes to genes linked to PD, helping scientists understand how these genes contribute to the disease. This research involves creating models that mimic human PD accurately and help identify potential treatments by testing drugs that might protect or restore dopamine neurons. Study shows that CRISPR and hPS cells can improve our understanding of PD and lead to better therapies. In the future, these technologies could lead to personalized treatments and curative cell replacement therapies for people with PD. This summary was initially drafted using artificial intelligence, then revised and fact-checked by the author.

## Introduction

Parkinson’s disease (PD) is one of the most age-dependent prevalent neurodegenerative disorders, affecting approximately 10 million people worldwide and imposing a growing clinical and economic burden as populations age^1–3^. Pathologically, PD is characterized by the progressive degeneration of dopamine (DA)-producing neurons in the substantia nigra pars compacta and the formation of Lewy bodies (LBs), which result from the aggregation of misfolded α-synuclein (α-syn), leading to debilitating motor symptoms. Current standards of care, most prominently DA replacement with levodopa, can provide temporary symptomatic relief. However, they neither halt disease progression nor address the underlying neuronal loss, and their efficacy invariably diminishes over time, whereas adverse effects accumulate^2–4^. Thus, it is essential to develop mechanism-directed strategies that move beyond symptomatic relief, together with precise human models that capture the molecular determinants of DA neuron vulnerability and regenerative approaches that replace or protect degenerating DA neurons. Conventional PD animal models, particularly those related to PD-linked toxin-induced and transgenic rodent models, have been crucial for elucidating the molecular and cellular mechanisms underlying the degeneration of DA neurons. However, these animal models insufficiently recapitulate key human-specific aspects of PD pathophysiology, including the progressive and selective degeneration of midbrain DA neurons and LB-like α-syn pathology. Such limitations underscore the necessity for human-relevant model systems that faithfully capture the complex molecular phenotype and temporal progression of PD^5^.

Human pluripotent stem cells (hPS cells), comprising human embryonic stem cells (hES cells) and human induced pluripotent stem cells (hiPS cells), offer a considerably promising cell source to compensate for the limitations of traditional animal models^6–10^, which often fail to yield limited translational success, potentially owing to different genetics and metabolism within species. hPS cells enable the directed differentiation of unlimited numbers of midbrain floor-plate-derived human DA neurons that exhibit human-relevant mitochondrial and lysosomal functions, α-syn aggregation, DA release and synaptic physiology^11,12^. These properties support mechanistic dissection, target validation and drug screening in a patient-matched genetic context. In parallel, hPS cell-derived DA cell products have emerged as a regenerative modality aimed at replacing lost DA neurons in PD^6^. Preclinical studies demonstrate that implanted DA cell products show long-range axonal innervation of the host striatum and behavioral rescue in PD animal models^13–18^. Clinical trials involving the transplantation of hPS cell-derived DA cell products into patients with PD are actively underway in several countries and have garnered considerable attention^19–22^.

Together with progress in hPS cell technology, the integration of gene-editing approaches, such as clustered regularly interspaced short palindromic repeats (CRISPR)–Cas9, permits precise introduction or correction of specific genetic variants and the programmable regulation of gene expression^23–25^. CRISPR enables the generation of wild-type, mutant and mutation-corrected lines, allowing phenotypes to be causally assessed for disease-related specific alleles within the identical genetic background. Beyond classical knock-in and knockout approaches, catalytically inactive deadCas9 (dCas9)-based systems, including CRISPR inhibition (CRISPRi) and activation (CRISPRa) and locus-targeted epigenome editors, expand the application to remodel chromatin and control the expression levels of disease-associated genes without double-strand breaks^26–29^. Furthermore, CRISPR technologies have been combined with high-throughput genetic and small-molecule screens to identify novel pathways related to disease progression^30,31^.

CRISPR-based editing has particularly provided precise hPS cell-based PD models that recapitulate pathological features in PD, including α-syn aggregation, mitochondrial dysfunction, autophagy dysfunction and loss of DA neurons. Representative targets involve *LRRK2*^32–34^, *SNCA*^29,35–37^, *PRKN*^38,39^, *PINK1*^40^ and *GBA*^41^. For example, the knock-in of LRRK2 G2019S in hPS cells reveals an impairment of the α-syn degradation pathway and neurite deficits, linking kinase hyperactivity to α-syn pathology^33^. *SNCA* dosage modulations, precise corrections and loss-of-function models in hPS cell-derived DA neurons define *SNCA* gene-dosage-dependent aggregation and toxicity and demonstrate the rescue of mitochondrial and electrophysiological phenotypes upon correction^35,36,42^. These PD models can also be applied to determine potential therapeutics, including protein aggregation inhibitors, mitochondrial modulators and autophagy enhancers. More advanced, dCas9 with transcriptional activators or inhibitors^26–28^ and genome-wide screens enable the identification of novel genes and pathways^30,31^ involved in PD progression and for developing strategies in an unprecedented manner. In addition, CRISPR-engineered reporter systems, such as tyrosine hydroxylase (TH)-linked fluorescent tags and lineage tracers, enable real-time visualization, purification and longitudinal tracking of DA neurons while preserving endogenous regulation, which is crucial for high-content phenotyping and enables the precise assessment of drug effects and screening^43–46^.

In addition to hPS cell-based PD models, hPS cell-based cell therapies for patients with PD have emerged as particularly promising strategies^6^. Despite remarkable progress in hPS cell-based cell therapy, hPS cell-derived grafts face several bottlenecks, including low post-grafting survival and the presence of residual heterogeneous or off-target contaminants^47–50^. CRISPR enables the identification of drivers of post-grafting cell death, reporter-guided enrichment of DA neuron identity and examinination of graft-directed functionality^51,52^. Taken together, advances in hPS cell technology and CRISPR engineering accelerate both the development of precise disease models and therapeutics in PD^53^, thereby overcoming the conventional limitations and increasing the translation potential^54^. This Review will discuss the past decade of progress in CRISPR-mediated strategies for hPS cell-based PD modeling and cell therapy development, outline the remaining challenges and provide future perspectives aimed at establishing the next generation of hPS cell-based disease models and cell replacement therapy in PD.

### CRISPR-mediated PD-relevant gene editing for hPS cell-based PD models

**Fig. 1:**
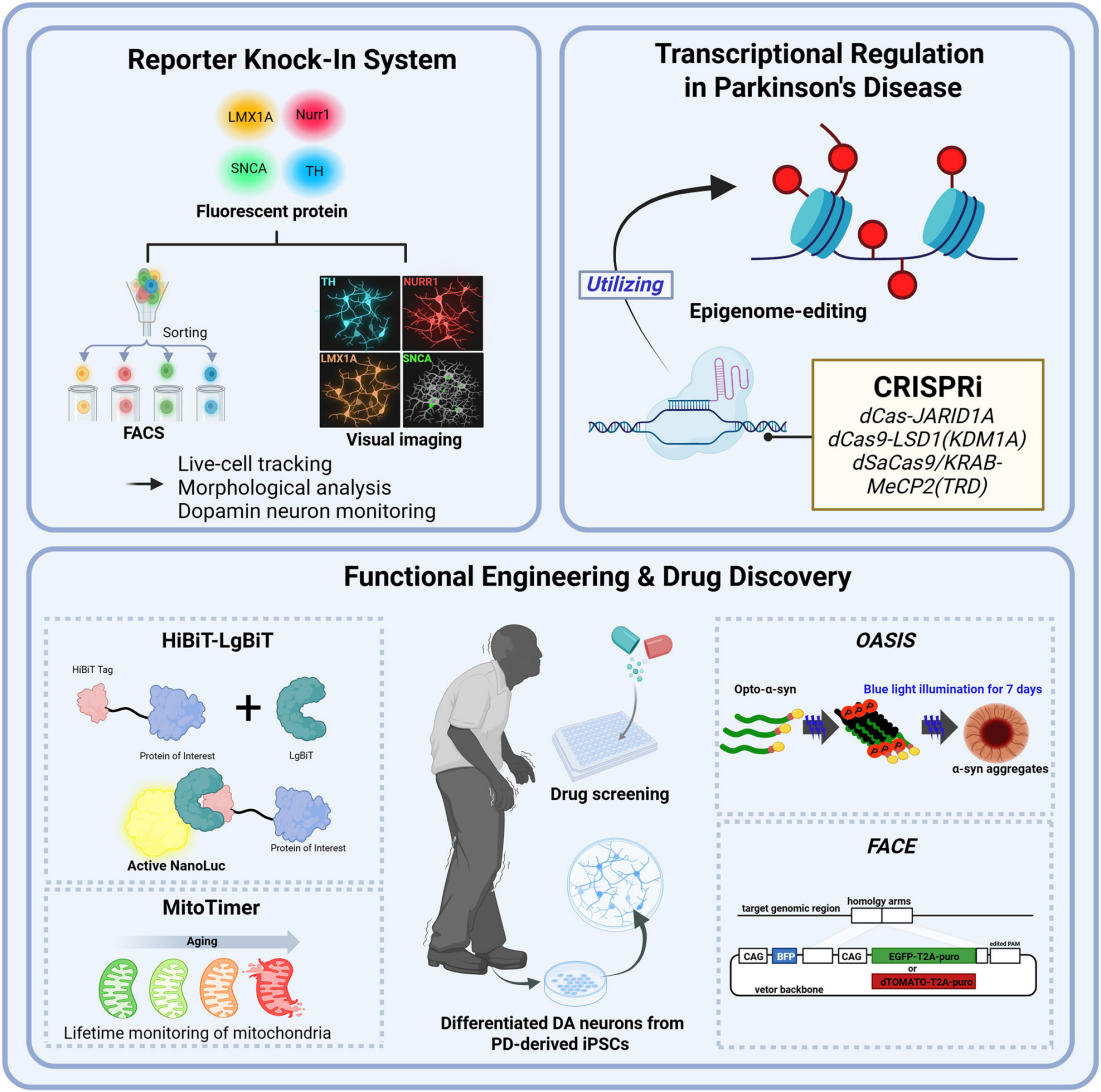
hPS cell engineering with CRISPR for PD Modeling and Drug Discovery. Top left: the endogenous fluorescent reporters are inserted at DA cell lineage- and disease-related loci (*LMX1A*, *NURR1*, *TH* and *SNCA*) to enable FACS purification, live-cell tracking, morphological analysis and monitoring of DA neurons. Top right: the epigenome-editing platforms, such as CRISPRi with [dCas-JARID1A, dCas9-LSD1(KDM1A), dSaCas9/KRAB-MeCP2(TRD)], modulate target genes implicated in PD, enabling causal studies of regulatory mechanisms. Bottom left: the assays include HiBiT–LgBiT NanoLuc for the sensitive quantification of tagged proteins and MitoTimer for lifetime mitochondrial tracking during cellular aging for drug screening using DA neurons derived from iPS cells derived from a patient with PD. Bottom right: the OASIS system (opto-α-syn) drives blue-light-inducible α-syn aggregation within a short period of time, whereas a targeted knock-in (FACE) enables the efficient insertion of fluorescent or selection reporters at specified genomic loci for controlled readouts for drug screening in PD-iPS cell-derived DA neurons. iPSC iPS cell.

CRISPR–Cas9 technology has rapidly emerged as an indispensable tool for modeling PD, enabling the precise perturbation or correction of PD-associated genes that converge on distinct yet interconnected cellular pathways implicated in DA neuron dysfunction and loss. PD-related genes are often grouped into functional categories such as mitochondrial quality control (*PINK1*, *PRKN*, *CHCHD2* and *RHOT1*)^38–40,55–58^, lysosomal and protein degradation pathways (*GBA1*, *DNAJC6* and *SYNJ1*)^59–62^, synaptic function (*SNCA*, *LRRK2*, *DNAJC6* and *SYNJ1*)^29,34,35,42,63–71^, neurodevelopment and neuroprotection (*Lin28A* and *PARK7*)^72,73^ and signal transduction and structural regulation (*LRP1*, *ZEB1* and *TSC2*)^74–76^. Targeting these categories recapitulates the hallmark of pathological phenotypes in hPS cell-based PD modeling, including mitochondrial dysfunction, impaired mitophagy, disrupted protein homeostasis, synaptic signaling defects and progressive DA neuron degeneration, thereby providing a causally tractable framework for dissecting disease initiation and progression (Table 1 and Fig. 1).

**Table 1:** CRISPR-based gene-editing strategies and related phenotypes in hPS cell-based PD models.

Gene	Edit type	Pre-edit background	How generated	Post-edit state (genotype)	Key phenotype (summary)	Reference(s)
*SNCA*	Copy-number tuning	Patient-derived iPS cell with compact *SNCA* quadruplication (4×)	CRISPR–Cas9 stepwise inactivation (indels/exon deletions)	Isogenic allelic series: 4× → 3× → 2× → 1× → 0× functional copies	α-Syn aggregates (spontaneous and seeded) increase with copy number; Ca²⁺ and mitochondrial defects; LB-like pathology in high copy lines	^29^
	Gene-dosage panel	Patient-derived iPS cell with *SNCA* triplication (3×)	CRISPR–Cas9 exon 2 frameshift	Isogenic dosage panel with graded α-syn levels	Resource for dose-dependent α-syn studies; enables expression/phenotype comparisons	^42^
	Correction	Patient-derived iPS cell (p.A53T)	CRISPR–Cas9 homology-directed repair	Wild-type *SNCA*	↓ α-Syn expression; rescue of mitochondrial function versus mutant	^35,105^
	Correction	Patient-derived iPS cell (p.A30P)	CRISPR–Cas9 homology-directed repair	Wild-type *SNCA*	↓ α-Syn expression; restored mitochondrial function	^63,64^
	Correction	Patient-derived iPS cell (triplication)	CRISPR–Cas9 homology-directed repair	Wild-type copy number	↓ α-Syn expression; ↓ aggregates	^65^
	Knockout	Wild-type hES cell	Cas9n; exon 2 deletion	*SNCA*^−/−^ (also ^+/−^)	Resistant to PFF-seeded pS129-α-syn (Lewy-like) pathology	^66^
	Knockout	Wild-type iPS cell	CRISPR–Cas9 exon 3 deletion	*SNCA*^–/–^	Resistance to MPP⁺ neurotoxicity	^67^
*LRRK2*	Point mutation knock-in	Wild-type iPS cell	CRISPR–Cas9 + piggyBac homology-directed repair (footprint-free)	G2019S knock-in	↓ Neuronal complexity; α-syn modulation changes	^68^
	Point mutation knock-in	Wild-type iPS cell	CRISPR–Cas9 homology-directed repair	G2019S knock-in	↑ α-Syn; chaperone-mediated autophagy defects	^69^
	Correction	Patient-derived iPS cell (G2019S)	CRISPR–Cas9 homology-directed repair	Wild-type *LRRK2*	↓ LRRK2 kinase activity; ↓ phosphorylated α-syn; normalization of ERK/transcriptional changes	^70,78^
	Knockout	Wild-type iPS cell	CRISPR–Cas9 exon 9 deletion	*LRRK2*^–/–^	Resource cell lines	^71^
	Variant panel (resource)	Wild-type iPS cell	CRISPR editing (multi-allele panel)	N1437H, R1441C/G/H, Y1699C, G2019S, I2020T, T1348N, K1906M; plus knockouts	Resource cell lines (panel for consistent isogenic comparisons across variants)	^34^
*PRKN*	Point mutation knock-in	Wild-type iPS cell	CRISPR–Cas9 homology-directed repair	p.A82E knock-in	↓ A9 DA pacemaking AP frequency	^39^
	Knockout	Wild-type iPS cell	CRISPR–Cas9 exon 2 deletion	*PRKN*^–/–^	Resource cell lines	^38^
*PINK1*	Correction	Patient-derived iPS cell (Q456X, I368N)	CRISPR–Cas9 homology-directed repair	Wild-type *PINK1*	↑ Mitophagy; ↑ TH⁺ neuron differentiation	^40^
	Knockout	Wild-type iPS cell	CRISPR–Cas9 exon 1 deletion	*PINK1*^–/–^	Resource cell lines	^38^
*CHCHD2*	Point mutation knock-in	Wild-type hES cell	CRISPR–Cas9 homology-directed repair	p.R145Q knock-in p.Q126X knock-in	MICOS/CHCHD10 perturbation → mitochondrial dysfunction	^56^
	Knockout	Wild-type iPS cell	CRISPR–Cas9 exon 1 deletion	*CHCHD2*^–/–^	↑ Mitochondrial respiration; ↓ ATP efficiency	^55^
*GBA1*	Mutation/correction (isogenic pairs)	Control/patient-derived iPS cell (N370S, E326K)	CRISPR–Cas9 homology-directed repair	N370S/E326K knock-ins and matched corrections	Resource cell lines; mTORC1–TFEB axis dysregulation Correction/activators: TFEB/ALP pathway restoration	^59,60^
*DNAJC6*	Point mutation knock-in	Wild-type hES cell	CRISPR–Cas9 homology-directed repair	Splice-site knock-in (c.801-2A→G)	α-syn accumulation; impaired midbrain DA development; mitochondrial and autolysosomal dysfunction	^61^
*Lin28A*	Point mutation knock-in/correction	Wild-type hES cell/R192G iPS cell	CRISPR–Cas9 homology-directed repair	p.R192G knock-in/wild-type iPS cell	Defects in midbrain DA development/maturation; ↑ α-syn oligomers rescue by wild-type LIN28A expression/correction	^72^
*LRP1*	Knockout	Wild-type iPS cell	CRISPR–Cas9 exon 6 deletion	*LRP1*^–/–^	↓ α-Syn (mono/oligo) uptake; reduced spread	^74^
*RHOT1* (Miro1)	Point mutation knock-in	Wild-type iPS cell	CRISPR–Cas9 homology-directed repair	R272Q, S156A, K572R knock-ins	Resource cell lines	^57^
	Correction	Patient iPS cell (T351A, T610A)	CRISPR–Cas9 homology-directed repair	Wild-type *RHOT1*	Resource cell lines	^58^
*PARK7*	Point mutation knock-in	Wild-type iPS cell	CRISPR–Cas9 homology-directed repair	p.A111L knock-in	Resource cell lines	^73^
*SYNJ1*	Point mutation knock-in/knockout	Wild-type iPS cell	CRISPR–Cas9 homology-directed repair or knockout	p.R258Q knock-in/*SYNJ1*^–/–^	↑ 1° cilia length, Cav1.3 and ubiquitin chain accumulation at ciliary base, presynaptic amphiphysin-2 clustering in DA neurons	^62^
*ZEB1*	Knockout	Wild-type hES cell	CRISPR–Cas9 exon 3 deletion	*ZEB1*^–/–^	↓ Neuronal differentiation/maturation	^75^
*TSC2*	Knockout	Wild-type iPS cell	CRISPR–Cas9 exon 37 deletion	*TSC2*^–/–^	mTORC1 hyperactivation in human neurons (↑ phospho-S6) after TSC2 exon 37 targeting	^76^
*SATB1*	Knockout	Wild-type hES cell	CRISPR–Cas9 knockout	*SATB1*^–/–^	Cellular senescence in DA neurons and impaired lysosomal and mitochondrial function	^79^

hPS cells, including PD-induced pluripotent stem cells (iPS cells), PD-derived DA neurons with CRISPR-based gene knockout or precise correction of pathogenic variants, enable the generation of isogenic control lines for direct phenotype comparison that differ only at the edited locus, thereby isolating mutation-specific effects within an identical genetic background. This approach facilitates precise mechanistic dissection of the contribution of individual genes to PD, spanning the early molecular events through late-stage neurodegeneration. Beyond faithfully reproducing disease phenotypes, these models provide a robust platform for linking specific molecular pathways to phenotypic outcomes, performing high-throughput drug screening and identifying therapeutic targets (Table 1 and Fig. 1).

*SNCA*, which encodes α-syn, is one of the most common causative gene mutations found in patients with PD. Physiologically, α-syn plays a role in the release of neurotransmitters. However, point mutations or gene multiplications (duplications or triplications) lead to aggregation of α-syn, forming LBs—a pathological hallmark of PD^29,37^. Leveraging CRISPR–Cas9, Iannielli et al. introduce targeted indels into each *SNCA* allele, creating an isogenic iPS cell panel with four to zero functional alleles. DA neurons derived from these lines were assessed for α-syn aggregation under normal and preformed fibril (PFF) treatment conditions, revealing gene-dosage-dependent increase in aggregation, with the quadruple copy of *SNCA* DA neurons (4× *SNCA*) showing the highest vulnerability^29^. In parallel, Zafar et al. edited exon 2 of patient-derived *SNCA*-triplication hiPS cells to generate multiple clones with distinct frameshift variants, enabling the analysis of how altered α-syn expression impacts physiological and pathological outcomes^42^. Given that gene correction studies enable mutation-specific PD modeling by removing pathogenic variants while preserving the identical genetic background, Barbuti et al. generated isogenic iPS cell clones from a patient with PD carrying the p.A30P *SNCA* mutation. Upon DA neuron differentiation, whereas uncorrected neurons exhibited impaired neuronal activity, reduced mitochondrial respiration, energy deficits and increased rotenone susceptibility, corrected clones showed reduced *SNCA* messenger RNA (mRNA) and protein levels^63^ and restored normal function^64^. Loss-of-function models further define α-syn-dependent seeding and toxicity. Using Cas9 nickase (Cas9n) to delete exon 2 of *SNCA* in hES cells generated *SNCA*^+/−^ and *SNCA*^−/−^ lines, their hPS cell-derived DA neurons resisted the formation of pS129-α-syn-positive aggregates after exposure to PFFs^66^. Similarly, Inoue et al. deleted exon 3 of *SNCA* in hiPS cells and resulting hiPS cell-derived DA neurons exhibited a loss of α-syn expression and reduced vulnerability to the DA neurotoxin 1-methyl-4-phenylpyridinium (MPP⁺)^67^. Collectively, CRISPR–Cas9-established *SNCA* dosage series, precise corrections and knockouts in hPS cells provide versatile platforms to interrogate α-syn’s roles in PD and to test targeted therapeutic options.

*Leucine-rich repeat kinase 2* (*LRRK2*) is a multidomain kinase involved in intracellular signaling and vesicular trafficking and a major genetic determinant implicated in both familial and sporadic PD^77^. The *LRRK2*-G2019S mutation is the most common pathogenetic *LRRK2* variant associated with PD^68^. Qing et al. combined CRISPR–Cas9 with piggyBac to produce a footprint-free, isogenic *LRRK2*-G2019S knock-in hPS cell. Functional analyses revealed that *LRRK2*-G2019S hPS cell-derived DA neurons displayed reduced neurite, fewer TH-positive cells and elevated α-syn phosphorylation (pS129)^68^. Extending the mechanistic study, Domenico et al. introduced the *LRRK2*-G2019S mutation into hPS cells and uncovered defects in chaperone-mediated autophagy, linking LRRK2 activity to degradative-pathway dysfunction that can potentiate α-syn pathology^69^. Altogether, these studies demonstrate that the *LRRK2*-G2019S mutation triggers structural and functional impairments in DA neurons, providing critical insights into the pathogenic mechanisms underlying PD development.

*PARKIN* (*PRKN*) encodes an E3 ubiquitin ligase, central to mitochondrial quality control and DA neurotransmission^39,46^. Similarly, the *PINK1* gene plays a pivotal role in maintaining mitochondrial function and morphology by recognizing and removing damaged mitochondria via mitophagy^40^. By CRISPR–Cas9 editing, Pu et al. introduced the PD-associated *PRKN* A82E mutation into hiPS cells derived from a healthy donor. Derived DA neurons exhibited a notable reduction in the frequency of pacemaking action potentials—an electrophysiological hallmark of DA neurons—functionally linking the variant to impaired electrophysiological homeostasis^39^. In complementary rescue work, Jarazo et al. corrected the Q456X and I368N mutations of *PINK1* in iPS cells from a patient with PD, which improved cellular metabolism, enhanced the DA neuron differentiation potential and reduced astrocyte enrichment, indicating that restoring the *PINK1*–*PRKN* axis normalizes key disease-relevant phenotypes^40^. These isogenic models directly connect mitochondrial function with DA neuron physiology and lineage commitment.

*Coiled-coil-helix-coiled-coil-helix domain containing 2* (*CHCHD2*) encodes a mitochondrial intermembrane space protein involved in mitochondrial oxygen consumption and apoptosis under oxidative stress^55,56^. An hPS cell with a deletion in exon 1 of *CHCHD2* and its derived DA neurons exhibited an increased proton leak and respiration, accompanied by reciprocal compensatory upregulation^55^. Furthermore, CRISPR-based engineered hPS cells harboring PD-associated *CHCHD2* mutations (R145Q and Q126X) yielded neural progenitors with mitochondrial dysfunction, reduced expression of CHCHD2 and MICOS complex and severe ultrastructural abnormalities in mitochondria characterized by near-complete cristae loss. These defects implicate CHCHD2 in the structural maintenance machinery of mitochondria and highlight it as a therapeutic target for restoring bioenergetic stabilization in PD^56^.

*RHOT1* (which encodes the tail-anchored protein GTPase MIRO1) is localized to the mitochondrial outer membrane, where it tethers mitochondria to molecular motors and is essential for mitochondrial transport. MIRO1 has been implicated in PD through its proposed interaction with PINK1 and Parkin^57^. Chemla et al. used CRISPR–Cas9 to correct T351A and T610A MIRO1 variants from iPS cells derived from a patient with PD, allowing the unambiguous attribution of transport and stress-response phenotypes to *RHOT1* dysfunction in human DA neurons^58^.

*GBA* encodes the lysosomal enzyme glucocerebrosidase, which breaks down glucocerebroside into glucose and ceramide^41^. This activity is crucial for proper lysosomal function and lipid metabolism. Mutations in *GBA* impair lysosomal function and autophagic flux, contributing to α-syn accumulation. hiPS cell-derived neurons from *GBA* mutation carriers show lysosomal dysfunction, increased α-syn levels and ER stress^41^. To investigate mechanisms underlying the pathological role of *GBA* mutations in PD, Mubariz et al. compared DA neurons differentiated from iPS cells from a patient with PD carrying the *GBA1* N370S mutation with CRISPR–Cas9-mediated corrected isogenic controls. The activity of TFEB, the master regulator of the autophagy–lysosomal pathway, was notably reduced in *GBA1*-mutant DA neurons, whereas no such alteration was observed in the gene-corrected cells^59^. These findings place TFEB-dependent transcription as a convergent deficit downstream of *GBA1* dysfunction and a rational target for pathway-level intervention.

*DNAJC6* encodes auxilin, a neuronal co-chaperone essential for clathrin-mediated endocytosis, and its mutation has been associated with juvenile-onset PD^61^. Wulansari et al. investigated the pathological role of *DNAJC6* in PD by using CRISPR–Cas9-mediated knock-in in hES cells to recreate the familial splicing-site mutation c.801-2A→G. The resulting human model displayed PD-relevant phenotypes, including mitochondrial dysfunction and impaired lysosomal function. These findings directly link endocytic defects to organellar stress and degradative failure in PD development^61^.

*Synaptojanin-1* (*SJ1*) encodes a phosphatidylinositol 4,5-bisphosphate (PI(4,5)P_2_) 4- and 5-phosphatase that is predominantly enriched in neurons and critical for removing endocytic factors during endocytosis^62^. The *SJ1* R258Q mutation selectively hinders its 4-phosphatase function and has been linked to PD. Using iPS cell-derived *SJ1* knockout and *SJ1* R258Q knock-in DA neurons with their isogenic controls established by CRISPR engineering, an abnormal accumulation of the calcium channel Cav1.3 and ubiquitin chains at the base of cilia, an area rich in SJ1, was observed, indicating the defective handling of ubiquitinated substrates and disrupted ciliary proteodynamics. These observations suggest that SJ1 is a key regulator of ciliary protein dynamics in DA neurons, with notable implications for DA homeostasis and PD pathogenesis^62^.

*LIN28A* is an RNA-binding protein that orchestrates fetal and early developmental programs and influences neuronal differentiation. Although it is not known as a high-risk PD-associated gene, LIN28 has been identified as a modifier of neuronal resilience^72^. The loss of *LIN28* function via the CRISPR–Cas9-mediated R192G substitution resulted in impaired cell morphology and induced abnormal cellular phenotypes, which are related to developmental defects and PD-linked features. Those defects were rescued by the re-expression of wild-type *LIN28A*, suggesting that early developmental regulators contribute to DA neuron vulnerability underlying PD pathogenesis^72^.

*Low-density lipoprotein receptor-related protein 1* (*LRP1*) is a transmembrane receptor belonging to the LDLR family that mediates and regulates the endocytosis of more than 30 ligands, including apolipoprotein E (APOE) and amyloid-β. CRISPR–Cas9 was used to delete exon 6 of *LRP1* in hPS cells, and the hPS cells were further differentiated into DA neurons. Relative to wild-type controls, LRP1-deficient DA neurons showed a markedly reduced internalization of monomeric and oligomeric α-syn, suggesting that LRP1 is a critical gatekeeper for neuronal α-syn entry^74^.

*ZEB1* is in the zinc finger E-box binding homeobox family and regulates cell morphological changes during epithelial–mesenchymal transition (EMT)^75^. In addition, ZEB1 is essential for the survival of neural stem cells in vitro. CRISPR–Cas9-mediated exon 3 deletion in *ZEB1* blocked the differentiation of hPS cell-derived neural progenitors into neurons. These findings define an essential role for ZEB1 in human neurogenesis and provide PD-relevant susceptibility pathways within a developmental context^75^.

*TSC2* mutations cause tuberous sclerosis complex, a neurological disorder characterized by intellectual disability and seizures, through the dysregulation of mTOR signaling, and they intersect with PD-related pathogenesis^76^. An hPS cell-knockout model with a deletion in exon 37 of *TSC2* via CRISPR engineering demonstrated hyperactivation of mTORC1 and hyperphosphorylation of its downstream target, ribosomal protein S6, which led to *TSC2*-knockout DA neuron vulnerability. These results highlight the utility of CRISPR–Cas9 for the mechanistic dissection of neurological disease phenotypes, even when the primary gene is not classified as a high-risk PD gene^76^.

*SATB1* is a nuclear chromatin organizer that has emerged as a DA neuron-enriched regulator of stress resilience in PD models^78^. In hPS cell-derived DA neurons, the CRISPR-based knockout of *SATB1* induced postmitotic DA neuron senescence phenotypes. Mechanistically, this reflected the derepression of a p21-driven senescence program^79^. A separate mechanistic study shows that a *SATB1*–*miR*–*22*–*GBA* axis links chromatin architecture to lysosomal lipid regulation and α-syn proteostasis, positioning SATB1 within the lysosome–autophagy pathway alongside GBA1^80^.

### CRISPR-mediated reporter systems for hPS cell-based PD models

Reporter knock-in systems in hPS cells have become essential tools for accurately identifying, isolating and longitudinally monitoring DA neurons in PD models. By integrating fluorescent cassettes into endogenous loci of DA neuronal lineage-related genes via CRISPR–Cas9, these systems enable real-time visualization, fluorescence-activated cell sorting (FACS) and high-content imaging while preserving native gene regulation. (Table 2 and Fig. 1).

**Table 2:** CRISPR technologies for establishing reporter systems and modulating gene expression in hPS cell-based PD models.

Category	Target	Strategy/tool	Locus/type	Objective	Results	References
Reporter system	*TH*	P2A–mOrange	Endogenous knock-in (*TH*)	Live imaging and FACS purification of TH⁺ DA neurons	Bright reporter enables live visualization; FACS isolation of TH⁺ neurons; functional Ca²⁺/electrophysiological readouts feasible	^44^
		T2A–mCherry		Couple TH identity to electrophysiology	Clear electrophysiological differences between TH⁺ versus TH^−^ populations; validates reporter specificity	^45^
		P2A–TdTomato		DN-specific readouts in isogenic PD/knockout contexts	↑ Oxidative stress across PD lines; selective DN loss in PARKIN-knockout; scalable 3D spin differentiation; FACS of TH⁺ neurons	^43^
		T2A–GFP		Label/enrich TH⁺ neurons to probe mitochondrial phenotypes	TH⁺ cells labeled via T2A–GFP cassette; enables EM/mitochondrial morphology analyses in PRKN-mutant DNs	^46^
	*NURR1*	2A–H2B-eGFP	Endogenous knock-in (*NR4A2/NURR1*)	Enrich/track midbrain DA (and as OASIS readout under opto-αSyn stress)	FACS yields near-pure NURR1⁺ midbrain DA; opto-α-syn reduces NURR1::GFP⁺ midbrain DA (vulnerability readout)	^79,81^
	*LMX1A*	LMX1A::Cre + AAVS1::Lox-STOP-Lox–BFP	Endogenous knock-in (*LMX1A*) + safe-harbor knock-in (*AAVS1*)	Lineage tracing/purification of midbrain DA progenitors; toxicity modeling	MPP⁺ preferentially depletes BFP⁺ (midbrain DA lineage) cells; lineage-specific assays enabled	^82^
	*SNCA*	α-Syn FLAG tag	Endogenous epitope tag (*SNCA*)	Visualize α-syn accumulation/aggregation dynamics	FLAG-α-syn puncta; aggregation monitoring in hPS cell-derived neurons	^69^
Gene regulation (CRISPRa/CRISPRi/epigenome editing)	*SNCA*	dCas9–VPR at *SNCA* TSS (CRISPRa)	Transcriptional activation (no cut)	Model α-syn overexpression in hPS cell-neurons; demonstrate bidirectional control with CRISPRi	↑ *SNCA* mRNA and protein in iPS cell-derived neurons	^83^
		dCas9–KRAB to *SNCA* TSS (CRISPRi)	Transcriptional repression (no cut)	Safely lower α-syn in human neurons	↓ *SNCA* mRNA/protein in iPS cell-derived neurons	^83^
		SunTag–JARID1A (H3K4me3 demethylation)	dCas9–SunTag–JARID1A at *SNCA*	Reverse pathogenic histone mark to reduce α-syn	↓ H3K4me3 at *SNCA*; ↓ α-syn levels	^26^
		Targeted DNA methylation editing	dCas9–DNMT3A to *SNCA* intron 1 CpG	Titrate promoter/intron methylation to lower SNCA	Targeted methylation editing → ↓ *SNCA*	^27^
		dSaCas9-based repression of *SNCA*	Catalytically dead *S. aureus* Cas9 (repression)	Reduce α-syn and associated stress	↓ α-Syn; ↓ mitochondrial DNA (mtDNA) damage/oxidative stress in patient-derived models	^84^
		Neuronal-type-specific CRISPRi	dSaCas9-KRAB/MeCP2, *TH*/*ChAT* promoter-driven	Cell-type-specific α-syn lowering in hiPS cell-DA/ChAT neurons	↓ *SNCA* mRNA/protein, ↓ pS129-α-syn, mitochondrial function recovery	^28^
Functional engineering	*SNCA*	OASIS (CRY2clust optogenetic α-syn clustering)	Knock-in readouts + optogenetic aggregation platform	Temporally/locally controllable PD-like stress; screening-ready	Light-induced α-syn aggregates; midbrain DA vulnerability; drug hits (for example, BAG)	^81^
	*SNCA*	FACE	CRISPR editing + FACS enrichment workflow	Rapidly enrich precise isogenic PD edits	Higher recovery of on-target *SNCA* edits; fewer byproducts	^85^
	*GBA1* (plus others)	HiBiT luminescent HCS lines	Small HiBiT tag + Dox-inducible master line (CRISPR-tagging)	Scalable protein quantification for PD genes/drugs	Robust Dox-inducible luminescence (for example, *GBA1*); compatible with HCS	^86^
	*COX8A*	MitoTimer KI iPS cell lines (AAVS1, Dox-inducible)	Safe-harbor *AAVS1* CRISPR knock-in (Dox-inducible MitoTimer)	Live tracking of mitochondrial turnover/dynamics	Quantifiable green → red shift maps mitochondrial age/turnover per neurite/cell	^87^
Screening platforms	*SNCA*	Sequential CRISPR screens	Genome-scale knockout/CRISPRi	Unbiased discovery of α-syn modifiers	Partial NatB inhibition lowers α-syn in iPS cell-derived neurons	^88^
	*PRKN*	PARKIN regulators screen	Genome-scale knockout (primary) + iPS cell-neuron validation	Map mitophagy/PRKN pathway regulators	*THAP11* identified; *THAP11* knockout ↑ pUb; validated in human neurons	^30^

One effective strategy for visualizing DA neurons is to faithfully track the expression of TH, a rate-limiting enzyme for DA synthesis. In this context, Calatayud et al. used a CRISPR–Cas9-based genome-editing strategy to knock-in the fluorescent protein mOrange into the last exon of the *TH* gene in hPS cells. Upon the differentiation of the *TH*–mOrange hPS cells into DA neurons, mOrange expression from the reporter colocalized accurately with endogenous TH expression. The FACS-based isolation of an mOrange-expressing hPS cell-derived cell yields homogeneous, viable DA neurons suitable for downstream physiological studies, including calcium imaging that distinguished DA neurons from non-DA neurons, providing a robust cellular model for studying PD phenotypes and facilitating high-content and high-throughput applications^44^. Independently, Rakovic et al. engineered a *TH*–mCherry reporter line that precisely overlapped with endogenous TH expression and demonstrated that TH⁺ neurons, relative to TH^−^ counterparts, exhibit larger sodium and potassium currents, stronger synaptic activity and higher frequency spontaneous action potentials. Importantly, the application of the floor plate differentiation protocol efficiently derived electrophysiologically mature TH⁺ dopaminergic neurons, underscoring the utility of this system for isolating PD-relevant neuronal subtypes and advancing high-content in vitro disease modeling^45^. Moreover, Ahfeldt et al. inserted a *TH*-P2A-TdTomato cassette into the endogenous *TH* locus and generated isogenic hPS cell lines harboring PD-associated gene mutations, including *PARKIN* (*PRKN*), *DJ-1* (*PARK7*) and *ATP13A2* (*PARK9*). *TH*–TdTomato reporters enabled FACS-based purification of DA neurons, live-cell tracking and three-dimensional (3D) morphological analyses that revealed gene-specific vulnerability. Only the *PARKIN*-deficient line exhibited marked DA-neuron loss upon differentiation, although all mutant lines showed increased oxidative stress^43^. Moreover, Yokota et al. created *TH*–GFP hPS cell lines from healthy donors and *PRKN*-mutant patients. TH⁺ *PRKN*-mutant DA neurons particularly displayed smaller, functionally compromised mitochondria when compared with TH⁻ counterparts, underscoring the mitochondrial vulnerabilities associated with PRKN-linked PD^46^. Collectively, these studies illustrate how *TH* reporter systems enable the isolation, functional characterization and mechanistic dissection of DA neurons in hPS cell-based PD models.

NURR1 is a transcription factor indispensable for DA neuron development and maintenance, expressed from the immature stage of the postmitotic DA neuron. Kim et al. established a *NURR1*–GFP reporter in optogenetically induced α-syn (opto-α-syn) PD-hPS cell lines to trace DA neurons. Blue-light-induced α-syn aggregation triggered the selective loss of NURR1⁺ cells, providing direct evidence for the selective DA neuron vulnerability to α-syn pathology^81^. In a separate study, Riessland et al. replaced the *NURR1* stop codon with *P2A-H2B-GFP* using CRISPR engineering, enabling the FACS-based enrichment of NURR1⁺ DA neurons and precise PD modeling and defining the pathogenic role of SATB1 in DA neuron senescence^79^. Together, *NURR1* reporters provide faithful readouts of postmitotic DA neuron identity, survival dynamics and PD-relevant stress responses.

Early lineage tracing and purification of DA progenitors are enabled by *LMX1A*-based reporter systems. Cardo et al. combined CRISPR–Cas9-mediated knock-in of P2A-Cre at the endogenous *LMX1A* locus with an *AAVS1* safe-harbor Lox-STOP-Lox–BFP reporter to establish an in vitro lineage-tracing and purification system. Following differentiation, immunocytochemical analysis and BFP tracing reveal that more than 60% of the cells were BFP^+^ and that 80–90% of LMX1A⁺ and BFP⁺ cells co-expressed FOXA2, confirming a strong correlation between BFP expression and midbrain DA neuron identity. In a PD-related toxin model, exposure to MPP⁺ resulted in a selective reduction of BFP⁺ (*LMX1A*-lineage) cells, demonstrating a higher vulnerability of DA neuron progenitors and mature DA neurons, an effect difficult to quantify without a lineage reporter^82^. This tracer system is therefore valuable for purifying early DA populations, quantifying lineage trajectories and testing how PD-relevant insults affect the fate and survival of DA neuron progenitors.

Beyond lineage reporters, CRISPR-based tagging systems to α-syn provide direct insight into α-syn proteostasis and cell-to-cell transfer. Using an hPS cell line bearing a CRISPR–Cas9-introduced FLAG tag at the *SNCA* locus, differentiated astrocytes exhibited abnormally elevated α-syn levels, with the precise co-localization of the tagged protein, facilitating the monitoring of accumulation and aggregation dynamics. Notably, a co-culture further revealed the transmission of FLAG-tagged α-syn produced in PD astrocytes into DA neurons, thereby providing direct evidence for astrocyte-to-neuron transmission of α-syn and highlighting a crucial non-cell-autonomous mechanism in PD pathogenesis^69^.

### CRISPR-mediated epigenome editing (CRISPRa, CRISPRi) for hPS cell-based PD models

CRISPR-based transcriptional regulation has become a central strategy in hPS cell models of PD. In particular, CRISPRi and CRISPRa, as well as locus-targeted epigenome editing, provide programmable, nuclease-free mechanisms to modulate gene expression with high precision when coupled to cell-type-specific promoters. Given that the expression level of *SNCA*, a key pathogenic determinant, is tightly linked to PD onset and progression, numerous studies have used CRISPRi to safely reduce α-syn levels by downregulating *SNCA* expression. These platforms preserve genomic stability while allowing dose-responsive and reversible control of gene expression, thereby clarifying the pathogenic mechanisms of dosage-dependent expression of PD risk genes and supporting the development of therapeutic strategies (Table 2 and Fig. 1).

#### CRISPRa and CRISPRi

Precise, bidirectional regulation of *SNCA*, the gene encoding the pathogenic protein α-syn, has become a central strategy for interrogating and potentially mitigating disease pathology. In hPS cell-derived DA neurons, transcriptional activation with dCas9-VP64/p65/Rta (VPR; *S. pyogenes*; dSpCas9-VPR) targeted to the SNCA transcription start site (TSS) increases α-syn mRNA and protein, whereas repression with dCas9-Krüppel-associated box (KRAB) (dSpCas9-KRAB) in the same system decreases both transcripts and protein. Thus, dCas9-based CRISPRa and CRISPRi enable the precise regulation of *SNCA* expression in hPS cell-derived DA neurons, providing a powerful platform to model pathogenic thresholds of *SNCA* levels and elucidate the specific contributions of *SNCA* dosage to cellular phenotypes relevant to PD^83^.

#### dSaCas9-based repression

To improve delivery and expand targetability, Sastre et al. engineered a compact CRISPRi platform based on *Staphylococcus aureus* dCas9 (dSaCas9), which is smaller than dSpCas9 and recognizes a distinct PAM (NNGRRT; KKH variant, NNNRRT)-fused to a KRAB repressor, enabling transcriptional repression of α-syn. A 32-single-guide RNA (sgRNA) screen identified a promoter-proximal sequence near the *SNCA* promoter, capable of modulating α-syn to defined expression levels. In hPS cell-derived DA neuron, the downregulation of α-syn reduced oxidative stress and decreased mitochondrial DNA damage, demonstrating a promoter-targeted strategy for direct transcriptional control of *SNCA*^84^.

#### Neuronal-type-specific CRISPRi

Sun et al. selectively targeted DA neurons implicated in PD and cholinergic neurons implicated in dementia with LBs^28^. To confer cell-type specificity, the dCas9-repressor vector was incorporated into the TH and choline acetyltransferase (ChAT) promoters, which are respectively active in DA and cholinergic neurons. To enhance the *SNCA* suppression, the synthetic transcriptional repressor KRAB/methyl CpG binding protein 2 (MeCP2) transcriptional repression domain (TRD) was introduced^28^. Applying this system to hPS cell-derived DA and cholinergic neurons derived from a patient with *SNCA* triplication resulted in the efficient and cell-type-specific downregulation of *SNCA* mRNA and protein, highlighting an efficient platform for a neuron-type-specific, *SNCA*-targeted epigenome-editing strategy.

#### Chromatin remodeling

In addition to repressor domains-based approaches, locus-specific chromatin remodeling strategies have been used to suppress *SNCA* expression. To determine whether the removal of H3K4me3 at the *SNCA* promoter alters α-syn expression, Guhathakurta et al. adapted a CRISPR–dCas9 SunTag-JARID1A system in which the JARID1A catalytic domain is recruited to the SNCA promoter^26^. The original SunTag platform, designed for up to ~300-fold transcriptional activation by recruiting multiple VP64 copies to a single-chain fragment variant (scFv) that binds GCN4, was modified by replacing VP64 with JARID1A to enable locus-specific demethylation. In PD iPS cell-derived DA neurons, this approach notably reduced H3K4me3 at the *SNCA* promoter and decreased α-syn levels, supporting a role for histone modifications at the SNCA locus and demonstrating that targeted chromatin remodeling can downregulate SNCA^26^. A complementary strategy utilized dCas9–DNMT3A to induce targeted DNA methylation within SNCA intron 1 in PD iPS cell-derived DA neurons, resulting in reduced SNCA mRNA and protein levels. Those studies highlight the therapeutic potential of programmable, repressive epigenome-engineering to repress dosage-dependent pathogenic expression without introducing double-strand breaks^27^.

These epigenome-editing platforms afford precise, reversible control. However, delivery efficiency, off-target chromatin effects and the durability of repression or activation over time remain key considerations for translational applications.

### CRISPR-mediated functional engineering for hPS cell-based PD models

Extending beyond CRISPRi, CRISPRa and epigenetic regulation, CRISPR technology has advanced toward functional engineering that reproduces pathogenic processes with spatial, temporal and quantitative control. Notable examples include (1) optogenetics-assisted α-syn aggregation induction system (OASIS) for the spatiotemporal control of protein aggregation^81^, (2) FACS-assisted CRISPR editing (FACE) for high-efficiency selection-based genome editing^85^, (3) HiBiT for sensitive protein quantification^86^ and (4) MitoTimer for real-time monitoring of mitochondrial protein function and dynamics^87^. Implementing these methods in hPS cell technology enables an increase in experimental resolution and provides human-relevant, scalable assays for offering mechanistic insights into disease progression and pathogenesis (Table 2 and Fig. 1).

#### OASIS

Kim et al. engineered an OASIS capable of rapidly inducing α-syn aggregation and neurotoxicity in PD hPS cell-derived DA neurons and midbrain organoids by fusing a blue-light-responsive domain, Cry2-clust, to mCherry-tagged α-syn. Cry2-clust induces homo-interaction upon light stimulation, enabling the precise control of aggregation dynamics^81^. The generation of opto-α-syn or an opto-mock knock-in in *SNCA* triplication hiPS cells via CRISPR–Cas9, and differentiation into DA neurons enables the spatiotemporal control of α-syn aggregation. Upon illumination, aggregates formed selectively in opto-α-syn neurons and were accompanied by increased cleaved caspase 3, a reduction in NURR1:GFP⁺ cells and loss of TH⁺ DA neurons, directly linking the controlled aggregation to cell death in human DA neurons. To discover small molecules that reduce aggregate formation, an OASIS knock-in line, combined with high-content imaging, supported a 1280-compound screen that yielded 19 primary hits, of which, 5 showed neuroprotective activity. In hPS cell-derived DA neurons containing opto-α-syn, drug candidates, including C-021 dihydrochloride (CDC) and BAG 956 (BAG), reduced 5G4⁺ aggregates and restored TH⁺ DA neuron survival in a dose-dependent and noncytotoxic manner. The effects are replicatively observed in opto-α-syn-midbrain organoids (reduced aggregates, improved survival). In the α-syn PFF-induced PD mouse model, the oral administration of BAG rescued TH⁺ DA neuron survival and improved motor, anxiety-related and cognitive behaviors. Collectively, OASIS provides a rapid, human-relevant platform to induce and quantify α-syn-linked toxicity across two-dimensional neurons and 3D organoids, supporting mechanistic studies and therapeutic screening.

#### FACE

Although CRISPR–Cas9 is routine for establishing isogenic disease and mutation-corrected lines, labor-intensive clonal screening to verify precise knock-ins and exclude random integrations or indels remains a bottleneck. To address this, Arias-Fuenzalida et al. proposed FACE^85^, in which donor vectors are engineered to contain two distinct modules, an internal positive selection module (for example, EGFP or dTomato) expressed only after precise, on-target integration within the homology arms and an external negative selection module (for example, tagBFP) expressed upon random, off-target integration. FACE implements ‘deterministic genotype editing’ by using FACS to resolve distinct fluorescent combinations, enabling the efficient isolation and expansion of biallelically edited cells without the need for labor-intensive clonal screening. Using FACE, isogenic *SNCA* A30P and A53T lines were generated, exhibiting altered energy metabolism consistent with phenotypes reported in differentiated neurons carrying the A30P variant. This validation demonstrates that FACE is a reliable and scalable method for deriving disease-relevant lines as well as a platform amenable to automated high-throughput PD phenotyping and drug discovery^85^.

#### HiBiT tag

Conventional fluorescent reporters, such as GFP, which is approximately 27 kDa, often disrupt the native protein function after tagging owing to their size. Split luciferase-based reporter systems overcome this limitation by providing high sensitivity and rapid quantification across a wide range of concentrations. Among these, HiBiT, a small 1.3-kDa peptide, tags endogenous proteins and binds to the complementary 18-kDa LgBiT subunit, which reconstitutes NanoLuc luciferase to produce a high-sensitivity luminescent readout^86^. Using an iPS cell line harboring a doxycycline-inducible LgBiT cassette inserted at the *CLYBL* safe-harbor locus, HiBiT tags were further introduced into eight PD-associated genes (*GBA1*, *GPNMB*, *LRRK2*, *PINK1*, *PRKN*, *SNCA*, *VPS13C* and *VPS35*), generating a panel of isogenic HiBiT fusion hPS cell lines. Functional validation showed strong doxycycline-dependent luminescence in the *GBA1* and *SNCA* HiBiT constructs. The pharmacological chaperone (ambroxol) treatment increased luminescence in *GBA1*-HiBiT hPS cell-derived DA progenitor cells compared with DMSO-treated controls and elicited a clear luminescent increase only in *GBA1*-HiBiT neurons^86^, providing a reliable, quantitative and real-time readout of endogenous PD risk gene products to elucidate PD mechanisms.

#### MitoTimer

MitoTimer is a fluorescent and bioluminescent biosensor that enables the real-time tracking of mitochondrial aging and renewal in live cells. A Timer protein targeted to the mitochondrial matrix via the *COX8A* signal sequence initially emits green fluorescence and gradually shifts to red as it matures. The observed color of MitoTimer reflects both the duration of Timer expression and the rates of mitochondrial protein incorporation and degradation. Thus, the red–green ratio reports mitochondrial protein turnover and biogenesis: higher ratios indicate older proteins (or reduced renewal), whereas lower ratios suggest newly synthesized proteins or increased mitophagy. Nadtochy et al. integrate MitoTimer into the *AAVS1* safe-harbor locus under the control of a doxycycline-inducible promoter in hPS cells, including two lines derived from FTDP-17 patients with PD carrying the pathogenic *MAPT* c.2013T → G (p.N279K) mutation and two from healthy donors. The hPS cell-derived DA neurons produced initial green fluorescence that progressively shifted to red, validating MitoTimer as a real-time readout for monitoring mitochondrial protein turnover in a disease-relevant context^87^.

#### Screening platforms

α-syn is closely associated with the pathogenesis of PD, yet the precise genetic networks that regulate endogenous αSyn (endo-αSyn) levels remain poorly defined. Kumar et al. established two α-syn reporter lines: (1) a melanoma cell line naturally expresses high α-syn, with a fluorescent tag inserted at the terminal *SNCA* exon, and (2) an hPS cell-derived neuronal reporter that quantifies endo-α-syn in neurons. Large-scale CRISPR-knockout and CRISPRi screens identified genes whose perturbation leads to reduced α-syn levels. Among these, enzymes of the N-terminal acetylation pathway, the NatB complex, emerged as the most potent negative regulators of α-syn levels across cell type^88^. Notably, the loss of N-terminal acetylation resulted in the rapid degradation of cytosolic α-syn^88^. In a complementary work on PARKIN that mediates the clearance of damaged mitochondria, Potting et al. performed a genome-wide CRISPR–Cas9-knockout screen in a cell line expressing GFP-PARKIN driven by the endogenous *PARK2* promoter to identify genes responsible for controlling PARKIN expression, given that the regulatory mechanisms controlling PARKIN expression and their influence on mitochondrial damage signaling remain poorly understood^30^. In total, 53 positive and negative regulators of PARKIN expression were identified, and among them, the functional validation of the transcriptional repressor, THAP11, demonstrated that the regulation of endogenous PARKIN directly modulates phospho-ubiquitin accumulation in multiple cell types, including *NGN2*-induced hPS cell-derived DA neurons, thereby linking PARKIN expression level to mitochondrial damage signaling and highlighting targets to restore mitophagy capacity^30^. These studies demonstrate that CRISPR-based screening platforms are powerful tools for systematically defining the genetic regulators of α-syn and PARKIN homeostasis and accelerating therapeutic target discovery in PD.

The efficiency and reliability of CRISPR–Cas9 editing in hPS cells are strongly influenced by the delivery strategy of the CRISPR-related cassette into hPS cells. Therefore, several approaches have been used, including electroporation, viral transduction and lipid-mediated systems. Each method balances editing efficiency, cell viability and biosafety considerations in distinct ways. A comparative overview of representative vectors, selection markers and contributing research groups is provided in Table 3.

**Table 3:** Transfection strategies for the use of CRISPR–Cas9 in hPS cells: vector, selection markers and delivery method.

Transfection method		Cell type	Vector/protein	Vector catalog	Source	Selection marker/sorting method	References
Electroporation	Neon transfection	iPS cell	pSpCas9n(BB)-2A-Puro (PX462) V2.0	Addgene no. 62987	Feng Zhang	Puromycin	^36^
		iPS cell	pSpCas9(BB)-2A-Puro (PX459) V2.0	Addgene no. 62988	Feng Zhang	Puromycin	^67^
		hES cell	pT7-Guide-IVT vector pT7-Cas9 vector	Origene		–	^56^
		iPS cell	CAG-Cas9-T2A-EGFP-IRES-Puro	Addgene no. 78311	Timo Otonkoski	Puromycin/GFP	^55^
		iPS cell	-	–		–	^74^
		iPS cell	pAC154-dual-dCas9VP160-sgExpression vector	Addgene no. 48240	Rudolf Jaenisch	–	^83^
		iPS cell	PX458 (pSpCas9(BB)-2A-GFP)	Addgene no. 48138	Feng Zhang	G418/puromycin	^87^
		iPS cell	Cas9 protein	–	–	mCherry sorting	^106^
	NEPA21	hES cell	pRGEN-CMV-Cas9-RFP-Puro	–	–	Puromycin	^61^
		hES cell	–	–	–	Puromycin	^72^
		iPS cell	pHL-EF1a-SphcCas9-iP-A	Addgene no. 60599	Akitsu Hotta	Puromycin	^46^
	MP-100	iPS cell	–	–	–	Puromycin	^70^
	Gene pulser Xcell	iPS cell	lentiCRISPR-V1-gRNA/puromycin selection cassette	Addgene no. 49535	Feng Zhang	GFP sorting	^33^
		hES cell	PX330	Addgene no. 42230	Feng Zhang	GFP sorting	^43^
	Nucleofector system (2b/2D/4D)	iPS cell	PX330S-2	Addgene no. 58778	Takashi Yamamoto	Neomycin	^45^
		hES cell	PX462	Addgene no. 62987	Feng Zhang	Puromycin	^66^
		iPS cell	PX459	Addgene no. 62988	Feng Zhang	–	^71^
		iPS cell	PX458	Addgene no. 48138	Feng Zhang	eGFP sorting	^81^
		iPS cell	PX330	Addgene no. 42230	Feng Zhang		^63,64^
		hES cell	PX330	Addgene no. 42230	Feng Zhang	GFP sorting/puromycin	^43^
		iPS cell	pSpCas9 (BB)-2A-GFP (PX458)	Addgene no. 48138	Feng Zhang	Puromycin	^39^
			Alt-R CRISPR–Cas9 crRNA and Alt-R CRISPR–Cas9 tracrRNA	Integrated DNA Technologies no. 1072533-		–	^34^
		iPS cell	PX330-U6-Chimeric_BB-CBh-hSpCas9	Addgene no. 42230	Feng Zhang	Puromycin	^57^
		iPS cell	BB-CBh-hSpCas9 (PX330)	Addgene no. 42230	Feng Zhang	Puromycin	^107^
		iPS cell	pCAG-puroRDTK·Neo		Yuet Wai Kan	Puromycin	^68^
			pCas9_GFP	Addgene no. 44719	Kiran Musunuru		
			gRNA_Cloning Vector	Addgene no. 41824	George Church		
		iPS cell	Alt-R CRISPR–Cas9 crRNA and Alt-R CRISPR–Cas9 tracrRNA	Integrated DNA Technologies no. 1072533-	–		^105^
		iPS cell	CAS9 protein	–		–	^60^
		iPS cell	pSpCas9(BB)-2A-GFP (PX458)	Addgene no. 48138	Feng Zhang	GFP sorting	^62^
		iPS cell	PX330A-1×2	Addgene no. 58766	Takashi Yamamoto	Puromycin	^82^
		iPS cell	PX330	Addgene no. 42230	Feng Zhang	Puromycin	^85^
Viral transfection	Lentivirus	iPS cell	pBK301	–	–	Puromycin	^27^
		iPS cell	LV-dCas9–DNMT3A-GFP/Puro, pBK301	–	–	Puromycin	^37^
		iPS cell	dSa- Cas9-p2a-puromycin			Puromycin	^28^
		iPS cell	pTRE-SadCas9-2xKRABdtTomato			tdTomato/BFP sorting	^84^
Lipid transfection	Lipofectamine stem/lipid transfection	iPS cell	Cas9 protein	–	–	Neomycin	^86^
		iPS cell	pSP-Ef1α-Cas9-2A-GFP	Integrated DNA Technologies		GFP sorting	^73^
		iPS cell	sgRNA-blast, spCas9-Puro			Puromycin, blasticidin	^29^
		iPS cell	PX458	Addgene no. 48138	Feng Zhang	GFP sorting	^31^
		iPS cell	CRISPR PiggyBac	Hera BioLabs		None	^42^
		iPS cell	CRISPR PiggyBac	Hera BioLabs			^35^
None lipid transfection	FuGENE	iPS cell	PX458	Addgene no. 48138	Feng Zhang	Geneticin	^69^
		iPS cell	pSpCas9(BB)-2A-GFP (PX458)	Addgene no. 12345	Feng Zhang	G418	^44^
Magnetofection	NeuroMag reagent	iPS cell	pCAG-dCas9-5xPlat2AflD	Addgene no. 82560	Izuho Hatada	–	^26^
			pHRdSV40-scFv-GCN4-sfGFP-VP64-GB1-NLS	Addgene no. 60910	Feng Zhang		
			lentiGuide-puro	Addgene no. 52963			

### CRISPR-mediated strategies for developing hPS cell-based cell therapy in PD

**Fig. 2:**
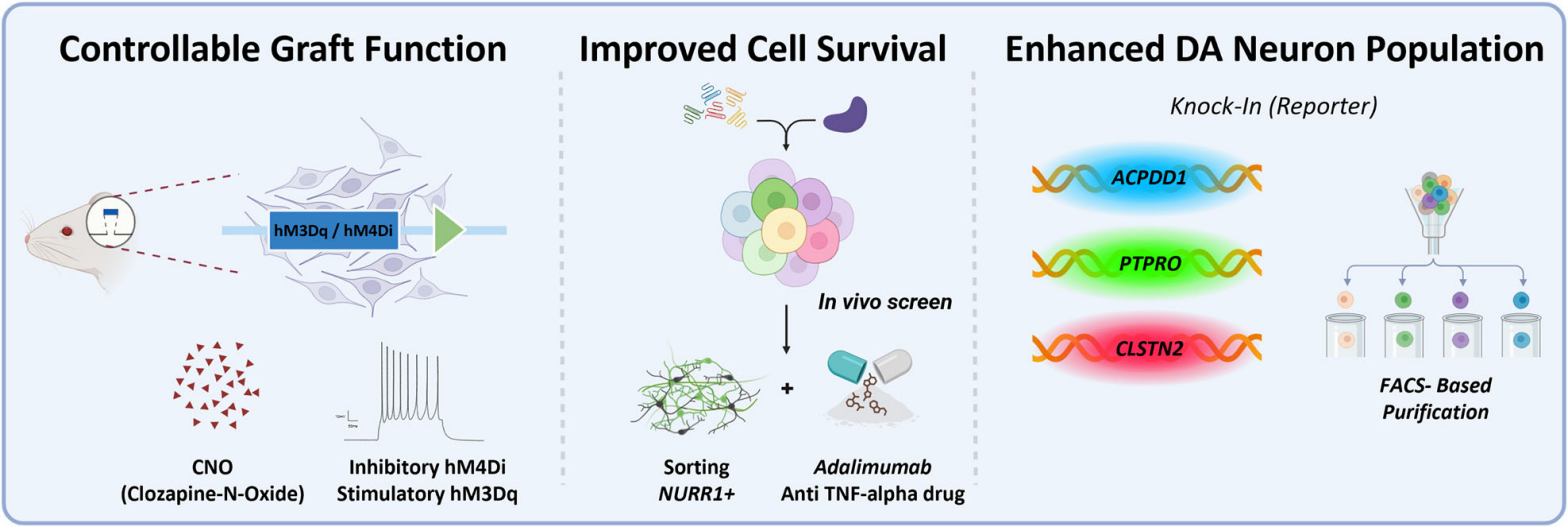
hPS cell engineering with CRISPR for cell replacement therapy in PD. Left: human DA grafts are engineered to express DREADDs with stimulatory hM3Dq or inhibitory hM4Di. A systemic CNO treatment enables the control of graft activity. Middle: the in vivo screen defines the restricted survival factor. Postmitotic DA neurons sorted with NURR1-driven GFP by FACS, and the administration of an FDA-approved drug, adalimumab (anti-TNF-α), can improve cell survival in the graft post transplantation. Right: the establishment of knock-in reporter lines targeting DA neuron lineage-related factors (APCDD1, PTPRO, CLSTN2) facilitates the FACS-based purification of reporter-positive cells, thereby increasing the fraction of the DA neuron population in vivo after transplantation.

Through defined patterning protocols that recapitulate midbrain floor-plate development, hPS cells can be efficiently directed to differentiate toward engraftable DA neurons^6,89^. Preclinical transplantation studies show that the grafts survive, extend long-range axons into the host striatum, release DA in an activity-dependent manner and restore motor functions^13–17^. First-in-human trials using clinical-grade, hPS cell-derived DA progenitors are now underway, and forthcoming safety and feasibility readouts are expected to guide the design of next-generation products and trials^19–22^. Despite progress in hPS cell-based cell replacement therapy in PD, major hurdles remain, including low post-grafting cell survival with more than 90% loss of transplanted DA neurons (both fetal and hPS cell-derived) observed and the risk of off-target cellular contamination within grafts (Fig. 2).

To address low post-grafting survival, Kim et al. conducted an in vivo CRISPR screen targeting 150 cell-death-related genes to identify the drivers of the loss of hPS cell-derived DA neurons in vivo after transplantation. An endogenous *NURR1*–GFP reporter was used to transplant postmitotic, NURR1⁺ DA neurons, thereby minimizing concerns about unwanted proliferation following gene knockout. The screen uncovered a cell-intrinsic TNF–NF-κB–p53 axis that triggers early post-grafting cell death. Importantly, the co-administration of the US Food and Drug Administration (FDA)-approved TNF inhibitor adalimumab with the graft markedly improved DA neuron survival and more effectively rescued PD-like behavioral deficits in a PD mouse model^47^.

In parallel, CRISPR was applied to generate reporter knock-ins by tagging fluorescent proteins at DA neuron lineage-related genes, such as *APCDD1*, *CLSTN2* and *PTPRO* loci. The FACS-based method to isolate these reporter-positive populations and implantation of the sorted cells increased the proportion of DA neurons and minimized off-target contaminants within the graft, thereby improving product consistency and quality control^90,91^.

Beyond survival and compositional purity in the grafted DA cell product, CRISPR enables the assessment of functional integration and plasticity of hPS cell-derived DA neurons after transplantation. To monitor and modulate integration and circuit engagement of hPS cell-derived DA neurons, a clozapine-*N*-oxide (CNO)-responsive designer receptors exclusively activated by designer drug (DREADDs) cassette was inserted via CRISPR knock-in, allowing the chemogenetic activation or inhibition of grafted cells^92^. With stimulatory hM3Dq or inhibitory hM4Di, this platform supports noninvasive, temporally precise control over the activation and inhibition of grafted cells, offering titratable behavioral rescue and potentially mitigating adverse effects.

### Future perspective for hPS cell-based model and cell therapy in PD

#### Integration of aging to hPS cell models

**Fig. 3:**
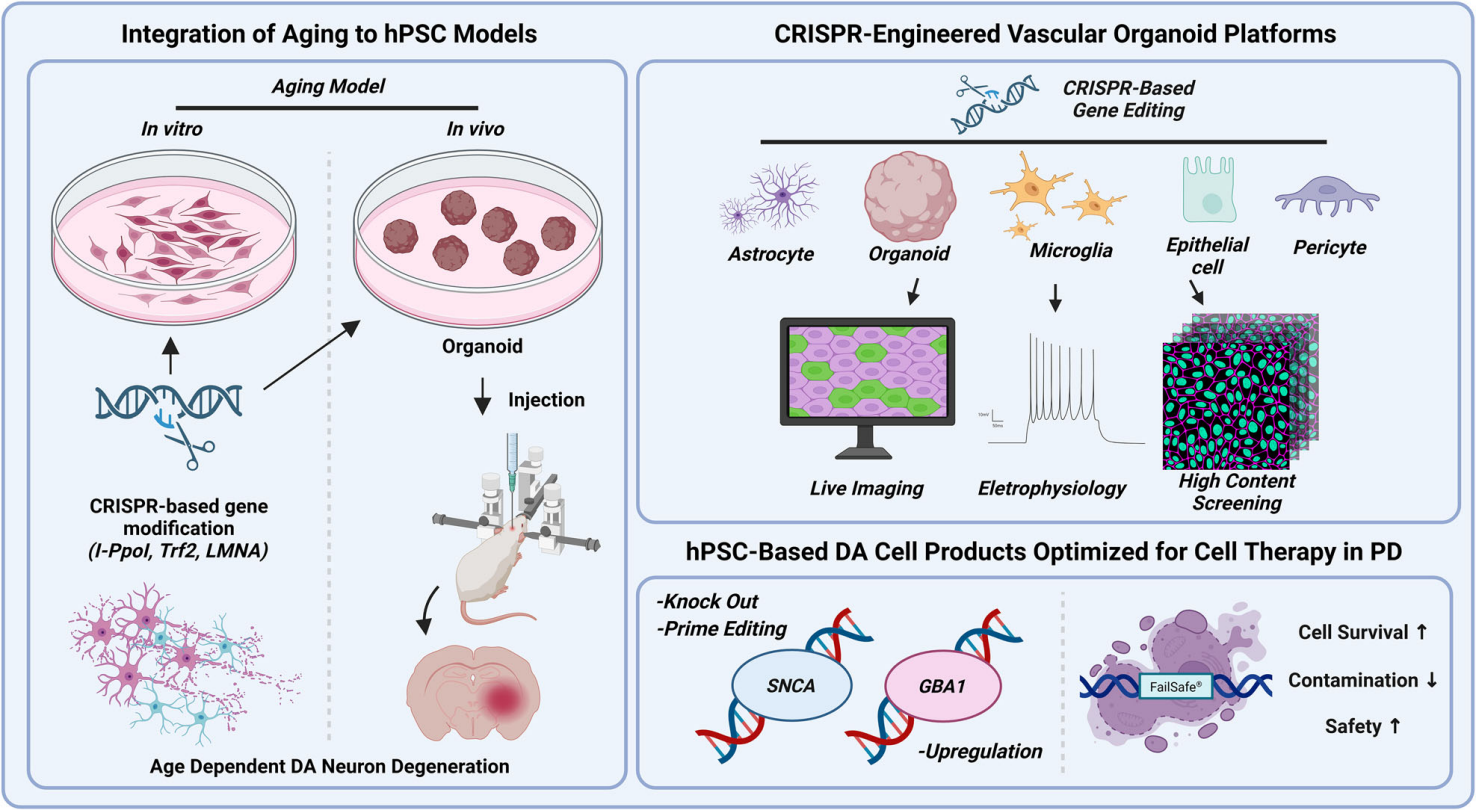
Future perspectives for hPS cell-based model and cell therapy in PD with CRISPR. Left: hPS cells combined with CRISPR-based gene editing of aging-related factors (*I-PpoI*, *Trf2* and *LMNA*) and aging models through the transplantation of aging-induced DA cells exhibit age-associated phenotypes, enabling age-dependent mechanistic studies in vitro and in vivo underlying PD onset. Top right: the CRISPR-based gene editing in a 3D organoid platform, consisting of brain cells, such as astrocytes, microglia and brain organoids, as well as endothelial cells and pericytes, enables multimodal readouts in improved physiological conditions- including live imaging, electrophysiology and high-content screening to discover modifiers of DA neuron vulnerability in a vascularized model. Bottom right: the targeted edits to reduce the PD pathogen (*SNCA* knockout) via prime editing and enhance lysosomal function (*GBA1* upregulation), paired with genetic fail-safe circuitry, to yield graft products with increased cell survival, reduced contamination and improved safety for hPS cell-based cell therapies in PD. hPSC hPS cell.

Although single, high-penetrance mutations (for example, *LRRK2*, *SNCA* and *PARKIN*) of hPS cell-based PD models provide valuable insights into the molecular mechanisms underlying PD development, sporadic cases of PD account for more than 90%, which are typically driven by a multifactorial interaction among polygenic risk variants, aging-related cellular decline and environmental influences^3^. Although these multifactorial factors underscore clinical heterogeneity and progression diversity observed in patients with PD, they remain underrepresented in current hPS cell-based systems. In particular, the conventional in vitro culture fails to fully recapitulate the aging-associated progression of cellular stress accumulation, mitochondrial decline, proteostasis collapse and cellular responses to environmental toxicity. As a result, current hPS cell-based models face fundamental challenges in recapitulating the pathophysiology of late-onset and sporadic PD. The limited availability of sporadic patient-derived materials further constrains model development. Accordingly, future studies may require combined strategies, such as CRISPR-mediated introduction of PD-associated mutations with targeted knock-in of progeric factors (for example, *I-PpoI*^93^, *Trf2*^94^ or *LMNA* variants^95^) to accelerate cellular aging in hPS cell-derived DA neurons. Such approaches may induce premature aging signatures and age-dependent pathology within hPS cell-derived DA neurons. Given the higher incidence of PD in aged individuals, aging models would enable a more physiologically relevant recapitulation of late-onset PD phenotypes, enabling the study of age-dependent DA neuron dysfunctions, including α-syn aggregation^96^, mitochondrial dysfunction^97,98^ and impaired proteostasis within a clinically relevant timeframe. Furthermore, advanced strategies such as long-term in vivo aging of transplanted CRISPR-edited human organoids^99^ or combined modeling approaches incorporating environmental stress and immune interactions could provide valuable platforms to investigate how aging and environmental factors simultaneously drive the development and progression of sporadic and late-onset PD (Fig. 3).

#### CRISPR-engineered vascular organoid platforms

The 3D organoids derived from hPS cells offer an advanced in vitro system to model PD-specific circuit dysfunction and cell to cell interactions^54,100,101^. Applying CRISPR–Cas9 to introduce or correct PD-associated variants in organoids, microglia, astrocytes and endothelial cells and pericytes enables the dissection of pathogenic mechanisms in a multicellular context, including neuron–astrocyte–microglia crosstalk, synaptic network alterations and cell-type-specific vulnerability in vascularized models. Moreover, coupling genome editing with live imaging, electrophysiology and high-content screening can accelerate the discovery of disease-modifying compounds and optimize gene- or cell-based therapies for clinical translation without reliance on animal models (Fig. 3).

#### hPS cell-based DA cell products to improve cell therapy in PD

Despite major advances in the efficacy of hPS cell-based cell therapies and in minimizing host immune responses, the long-term prevention of graft rejection remains a major unresolved challenge^49,50^. The next generation of hPS cell-derived DA cell products may therefore embed CRISPR-enabled improved safety, survival and function directly into the graft. First, the selective elimination of non-DA cell contaminants, such as drug-inducible expression and fail-safe ‘suicide’ switch systems designed using CRISPR, can remove residual nontarget lineages, including proliferative cells, thereby improving safety in hPS cell-based cell therapy for PD. Next, to mitigate the transplantation-associated inflammation and physical damage associated with the transplantation procedure, immune-tolerizing gene-cell strategies using CRISPR knock-ins, such as the transplantation of DA cells engineered to express ligands that attenuate T-cell/NK/complement activity, could enhance both survival and functional integration while reducing chronic immune responses. In addition, anticipating the host-to-graft propagation of α-syn, pathology-resistant designs, such as CRISPR-mediated *SNCA* knockout and dose reduction or upregulation of α-syn-degrading pathways (for example, *GBA1*), offer a rational option to long-term graft functionality. In particular, the recent emergence of base and prime editing offers precise, double-strand break-free strategies to engineer these functional modifications, highlighting the ongoing evolution of genomic tools for enhanced neuronal differentiation and the potential application of CRISPR-based therapeutic approaches to improve cell replacement therapy with increased safety in the clinic^102,103^. Together, these strategies outline a road map toward safer, immune-adapted and disease-resilient hPS cell-derived DA cell products for PD (Fig. 3).

## Conclusion

Advances in CRISPR and hPS cell technologies enable precise, mechanism-level PD modeling within the same genetic background, as well as the optimization of manufacturable DA cell products. CRISPR-based isogenic edits, CRISPRi, CRISPRa and locus-specific epigenome control combined with large-scale screens enable tractable studies across organellar stress, synaptic failure, α-syn proteostasis, mitochondrial, lysosomal and immune pathways in hPS cell-based PD models. In hPS cell-based cell therapy in PD, CRISPR engineering enables enriched survival (in vivo screen), homogeneous (the reporter-based enrichment), safer (inducible fail-safes), controllable (chemogenetic control), immune-adapted (hypo-immunogenic edits) and pathology-resistant (*SNCA* downtitration and *GBA1* upregulation) to overcome acute cell death, contamination, graft rejection and long-term prevention of α-syn propagation in grafts. In this regard, CRISPR-enabled hPS cell platforms offer valuable insights for moving PD therapeutics beyond symptomatic relief toward mechanism-based, scalable and durable disease-modifying interventions. Despite the advantages of CRISPR-based hPS cell platforms, the clinical translation of CRISPR-engineered hPS cell products requires the careful consideration of ethical and regulatory aspects, including the responsible use of genome editing, long-term genomic stability and effective management of off-target risks. Ensuring ethically compliant genome engineering practices is essential for maximizing the safe therapeutic outcome^104^.
